# Efflux in Fungi: La Pièce de Résistance

**DOI:** 10.1371/journal.ppat.1000486

**Published:** 2009-06-26

**Authors:** Jeffrey J. Coleman, Eleftherios Mylonakis

**Affiliations:** Division of Infectious Diseases, Massachusetts General Hospital and Harvard Medical School, Boston, Massachusetts, United States of America; University of British Columbia, Canada

## Abstract

Pathogens must be able to overcome both host defenses and antimicrobial treatment in order to successfully infect and maintain colonization of the host. One way fungi accomplish this feat and overcome intercellular toxin accumulation is efflux pumps, in particular ATP-binding cassette transporters and transporters of the major facilitator superfamily. Members of these two superfamilies remove many toxic compounds by coupling transport with ATP hydrolysis or a proton gradient, respectively. Fungal genomes encode a plethora of members of these families of transporters compared to other organisms. In this review we discuss the role these two fungal superfamilies of transporters play in virulence and resistance to antifungal agents. These efflux transporters are responsible not only for export of compounds involved in pathogenesis such as secondary metabolites, but also export of host-derived antimicrobial compounds. In addition, we examine the current knowledge of these transporters in resistance of pathogens to clinically relevant antifungal agents.

## Introduction

Members of the fungal kingdom are found in almost all habitats and exist as saprobes, commensals, and pathogens. Approximately 11,000 plant diseases have been attributed to fungi in over 120 genera [1], while in the clinical setting, the number of invasive fungal infections has increased steadily over the past two decades. *Candida* sp. is the fourth most common pathogen isolated from blood cultures [2], and other pathogens such as *Cryptococcus* sp., *Aspergillus* sp., *Fusarium* sp., and zygomycetes, have an unacceptably high morbidity and mortality [3]–[5]. In addition, the incidence of mycoses caused by opportunistic fungi is rising [6].

The diverse nature of fungi can be attributed to such factors as increased capacity to utilize a wide range of carbon and nitrogen sources, the capability for rapid growth, and the ability to adapt to otherwise harsh environments. Fungi are constantly bombarded by toxic compounds from external sources. These compounds can be synthesized by other microorganisms to impede the fungus for a competitive advantage to limited resources, synthesized by hosts as a defense mechanism, or may be present in the environment. Regardless of the source, these toxic compounds force the fungus to evolve mechanisms in order to survive. One common method to overcome these antifungal compounds is active efflux, which prevents any intracellular build-up of the compound and, therefore, renders the fungus resistant or tolerant to the otherwise toxic compound.

As research in mycology progresses it is becoming evident that transporters are important factors in pathogenicity. Several transporters have been described as being involved in fungal pathogenicity, including a range of cellular processes such as calcium entry, vescicle transport, stress tolerance, dimorphic switching, capsule synthesis, iron acquition, and virulence factor enzyme activity [7]–[16]. This review will focus on the ATP-binding cassette (ABC) transporters and the major facilitator superfamily (MFS) transporters associated with secondary metabolites, such as mycotoxins, and resistance to natural toxic compounds or antifungal drugs, which have a proven or implicated role in fungal pathogenesis (Table 1).

**Table 1:** Summary of Transporters Involved in Virulence or Toxin and Drug Efflux.

Transporter Family	Name	Fungus	Description/Substrate	Amino Acid Length	References
**ABC Transporters**					
PDR/ABCG	CDR1	*C. albicans*	Triazole resistance	1500	[60],[72]
PDR/ABCG	CDR2	*C. albicans*	Triazole resistance	1499	[71]–[73]
PDR/ABCG	CgCDR1	*C. glabrata*	Fluconazole, itraconazole	1542	[59]
PDR/ABCG	CgCDR2/PDH1	*C. glabrata*	Fluconazole, ketoconazole	1499	[61],[85]
PDR/ABCG	CgSNQ2	*C. glabrata*	Fluconazole, itraconazole	1507	[62]
PDR/ABCG	AFR1	*C. neoformans*	Triazole resistance	1543	[58]
PDR/ABCG	**ABC1**	*M. grisea*	Unknown function	1619	[46]
PDR/ABCG	**GpABC1**	*G. pulicaris*	Rishitin tolerance	1491	[51]
PDR/ABCG	**Mgatr4**	*M. graminicola*	Unknown function	1635	[49]
PDR/ABCG	**BcatrB**	*B. cinerea*	Resveratrol tolerance	1439	[48],[50]
MRP/ABCC	**MLT1**	*C. albicans*	Unknown function, possibly bile	1606	[55]
**MFS Transporters**					
DHA14	TOXA	*C. carbonum*	HC-toxin secretion	548	[34]
DHA14	TRI12	*F. sporotrichioides*	Trichothecene T-2 secretion	598	[40]
DHA14	**CFP**	*C. kikuchii*	Cercosporin secretion	607	[35]
DHA12	**CTB4**	*C. nicotianae*	Cercosporin secretion	512	[36]
DHA12	cefT	*A. chrysogenum*	Cephalosporin secretion	561	[42]
DHA14	**PEP5**	*N. haematococca*	Unknown function	592	[52]
DHA12	MDR1	*C. albicans*	Fluconazole resistance	564	[76]–[78]
DHA12	FLU1	*C. albicans*	Fluconazole resistance	610	[79]
DHA12	CdMDR1	*C. dubliniensis*	Fluconazole resistance	557	[80],[81]
DHA12	TMP1	*C. albicans*	MDR	561	[83]
DHA12	TMP2	*C. albicans*	MDR	581	[83]

Transporters involved in virulence are in bold.

## Overview of Efflux Pumps

The two most extensively studied families of transporters involved in efflux are the ABC transporters and the MFS transporters. Fungi dedicate a large amount of their genome to encoding transporters, as there are approximately ten to 30 genes encoding transporters per megabase of genomic DNA in fungal genomes [17]. The most common type of transporter in all sequenced fungal genomes to date is the MFS transporter. However, members of the ABC transporter superfamily are the most common of the primary transporters (Table 2). Together, these two superfamilies account for approximately half of all the genes encoding transporters in fungal genomes. Despite the importance of these two families of transporters in virulence, there is no apparent correlation between the quantity of these transporters in fungal genomes and the pathogenicity of the fungus (a saprobic isolate versus a pathogenic isolate). For example, *Aspergillus nidulans* and the closely related human pathogen *Aspergillus fumigatus* both have 45 ABC transporters encoded in their genomes, and *A. nidulans* has more MFS transporters than *A. fumigatus* (Table 2). Also of note, the group known as oomycetes, although not members of the fungal kingdom, but sometimes loosely grouped with fungi, have 4- to 5-fold more ABC transporters than true fungi; however, this difference could be due to the larger genome size of the oomycetes.

**Table 2:** Number of ABC and MFS Transporters in Sequenced Fungal and Oomycete Genomes.

Classification	Fungus	ABC^a^	MFS
**Oomycetes**	*Phytophthora infestans*	160	102
	*Phytophthora ramorum*	185	113
	*Phytophthora sojae*	190	109
**Chytridiomycota**	*Batrachochytrium dendrobatidis*	52	23
**Zygomycota**	*Rhizopus oryzae*	44	111
**Basidiomycota**	*Ustilago maydis*	38	91
	*Puccinia graminis*	26	35
	*Cryptococcus neoformans* JEC21	29	192
	*C. neoformans* H99	32	159
	*Coprinus cinereus*	47	95
	*Phanerochaete chrysosporium*	56	113
**Ascomycota**			
Taphrinomycotina	*Schizosaccharomyces pombe*	9	58
Saccharomycotina	*Saccharomyces cerevisiae*	24	85
	*Candida albicans* SC5314	21	85
Eurotiomycetes	*Aspergillus nidulans*	45	356
	*Aspergillus fumigatus*	45	275
	*Penicillium chrysogenum*	51	416
	*Coccidioides posadasii* C735	30	146
	*Paracoccidioides brasiliensis* Pb18	28	103
	*Histoplasma capsulatum*	33	86
	*Microsporum gypseum*	45	178
Dothideomycetes	*Stagonospora nodorum*	46	301
Leotiomycetes	*Sclerotinia sclerotiorum*	44	169
	*Botrytis cinerea*	51	204
Sordariomycetes	*Magnaporthe grisea*	50	251
	*Chaetomium globosum*	41	155
	*Neurospora crassa*	31	141
	*Gibberella zeae* (*Fusarium grmainearium*)	56	335

The number of transporters in each genome was calculated from either the genome publication (*P. chrysosporium*, *P. chrysogenum*), the TransporterDB (*P. infestans*, *P. ramorum*, *P. sojae*, *C. neoformans* JEC21, *S. pombe*, *S. cerevisiae*, *A. nidulans*, *A. fumigatus*, *C. posadasii*, *N. crassa*) or from searching the Broad Fungal Genome Initiative Web site (http://www.broad.mit.edu/node/304) for the PFAM domains corresponding to the transporter.

a The number of ABC transporters from genomes from the Broad Fungal Genome Initiative includes the number of ABCE and ABCF proteins (usually 4–5 members), as they contain the conserved sequence; however, they are not transporters.

Members of the ABC transporter superfamily are primary efflux transporters, which, as their name implies, hydrolyize ATP for export of the substrate. This superfamily is further broken into five families of transporters (ABCA, ABCB, ABCC, ABCD, and ABCG), of which three families are involved in efflux of toxic compounds. Members of these three families, ABCB, ABCC, and ABCG, also referred to as the multi-drug resistance (MDR), multi-drug resistance–associated protein (MRP), and the pleiotropic drug resistance (PDR) families, respectively, have been extensively studied in *Saccharomyces cerevisiae*, providing insight into their possible functions in pathogenic fungi [18],[19]. Of the ABC transporter families, the PDR family has the least amount of phylogenetic conservation, demonstrating both gene loss and duplication within yeasts [20] and filamentous fungi, which suggests that members of this family are rapidly evolving from outside selective pressures.

**Figure 1:**
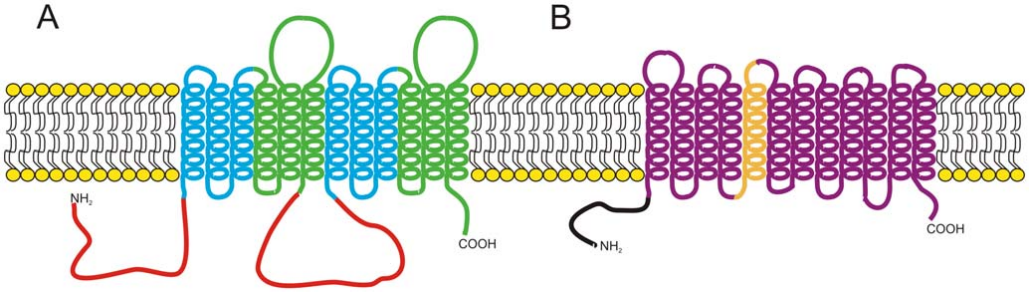
Schematic representation of the two main fungal transporters responsible for efflux of toxic compounds. Representation of an ABC transporter of the PDR family (A) and an MFS transporter of the DHA12 family (B). The NBDs of the ABC transporter are depicted in red, while the TMDs important for substrate specificity are colored green and gold for the ABC and MFS transporters, respectively.

The conserved architecture of ABC transporters is composed of a nucleotide-binding domain (NBD) followed or preceded by six transmembrane-spanning helices creating a transmembrane domain (TMD). These two domains together represent the NBD-TMD_6_ design of half ABC transporters, which dimerize to form a fully functional protein [21]. Instead of dimerizing, most fungal ABC transporters have evolved to be comprised of two fused NBD-TMD_6_ half transporters, creating one functional protein (Figure 1) [21],[22].

The MFS transporters are smaller in size compared to the ABC transporters since they do not require an NBD; however, they still contain 12 or 14 transmembrane-spanning helices (Figure 1). Translocation of the substrate is driven by the proton gradient generated across the plasma membrane [23]. This family is divided into 17 families, of which two, the drug:H^+^ antiporter (14 spanner) (DHA14) and the drug:H^+^ antiporter (12-spanner) (DHA12), are involved in efflux of toxic compounds [23].

## Transporters in Plant Pathogens

Virulence-associated efflux pumps in plant pathogenic fungi can be classified into two categories. The first class of transporters is responsible for secretion of virulence factors or compounds produced by the fungus. Several fungi synthesize and secrete low molecular weight compounds that are not required for growth. These compounds, termed secondary metabolites, are bioactive and are sometimes involved in virulence [24],[25]. Secondary metabolites are commonly synthesized by clusters of genes that usually include either a polyketide synthase (PKS), nonribsomal peptide synthase (NRPS), or a fusion of both enzymes (PKS-NRPS), which is responsible for the majority of the synthesis of the metabolite [24],[25]. Other accessory proteins within the gene cluster are involved in the secondary metabolite's biosynthesis, and frequently one (or more) gene(s) encoding a transporter is associated with these secondary metabolite gene clusters. Examples of secondary metabolites are the iron sequestering compounds, termed siderophores [26],[27], which are virulence factors for a number of pathogens [28]–[30]; mycotoxins such as penicillin G, gliotoxin, aflatoxin, and lovastatin; and the host-specific and -nonspecific toxins such as T-toxin, victorin, botrydial, AF-toxin, and cercosporin [31].

Phytotoxins produced by fungi that are involved in virulence on a specific host plant, and therefore expand host range, are termed host-specific toxins (HSTs), and while not all plant pathogens produce HSTs, they are important virulence factors for members of the *Cochliobolus* and *Alternaria* species [31],[32]. For example, the phytopathogenic fungus *Cochliobolus carbonum* produces a cyclic tetrapeptide HST, termed HC-toxin, which functions as an inhibitor of histone deacetylases in several organisms and is a virulence factor on maize [33]. The synthesis of this compound is carried out by an NRPS named HTS1 [33], and the locus responsible for the synthesis of this compound contains two genes encoding MFS transporters, termed *TOXA* and *TOXB*, postulated to be responsible for efflux of HC-toxin into the host plant cell [34]. In another example, the plant pathogenic *Cercospora* species produces a host-nonselective toxin, cercosporin, which is also synthesized by a secondary metabolite gene cluster. Cercosporin is exported out of the fungal cell by the cercosporin facilitator protein (CFP) in *C. kikuchii* and by CTB4 in *C. nicotinae* [35],[36]. These 12-membrane-spanning MFS transporters appear to be orthologous, and mutants of these genes are reduced in virulence on their host plants. However, the rice pathogen *Magnaporthe grisea* produces a secondary metabolite via the PKS-NRPS fusion protein termed ACE1 and the surrounding accessory proteins. This cluster contains a gene encoding an MFS transporter (MFS1), although it is not involved in efflux of the ACE1 metabolite as it has a deletion of a single base pair resulting in an early stop codon [37]. Therefore, the ACE1 metabolite must rely on another transporter encoded outside the secondary metabolite gene cluster for export.

Efflux pumps in secondary metabolite gene clusters that are responsible for synthesis of mycotoxins are less well understood. The expression of MFS transporters in mycotoxin secondary metabolite clusters appears not to be controlled by the same transcription factors as the rest of the secondary metabolite cluster but, instead, is induced by the accumulation of the synthesized metabolite [38],[39]. However, the ABC transporter TRI12 in the trichothecene T-2 toxin cluster contains the conserved sequence in the promoter region for binding of the cluster's transcription factor, suggesting it is under the same control as the rest of the gene cluster [40]. Mutants of efflux pumps in secondary metabolite clusters have been constructed, and most of these mutants retain wild-type levels of mycotoxin secretion [38],[39],[41]. However, studies where the efflux pump was expressed in a homologous system or increased in copy number resulted in either efflux of the compound or a corresponding increase in efflux [40],[42], demonstrating that the transporters are actually involved in efflux of the mycotoxin. The most obvious reason why mutants of transporters in secondary metabolite clusters lack a reduction of toxin secretion is due to other transporters with an overlapping substrate range. Supporting this notion, mutants of the ABC transporter *atrD* in *Aspergillus nidulans*, although not in a cluster, produce less penicillin than the wild-type, suggesting this transporter is involved in efflux of the compound [43].

The second class of transporters involved in plant virulence is responsible for efflux of molecules produced by the host plant. As a defense mechanism, plants produce antimicrobial compounds. These low molecular weight compounds, termed phytoalexins or phytoanticipins depending on when they are synthesized, have diverse chemical structures. Over 25 years ago, studies conducted on *Nectria haematococca* MPVI and the pea phytoalexin pisatin suggested that a fungal efflux mechanism is involved in the tolerance to this plant-derived defense compound and, therefore, may be a pathogenicity factor [44],[45]. However, it was not until 15 years later that the first fungal ABC transporter (ABC1) was demonstrated to be a virulence factor in the phytopathogenic fungus *M. grisea*, the causative agent of rice blast disease [46].

Since that time a number of ABC transporters have been identified as virulence factors on host plants. Most of these transporters are members of the PDR family of ABC transporters (Table 1). A number of phytoalexins and other toxic compounds induce expression of these fungal transporters [46]–[50]; however, only a few have demonstrated the ability to confer tolerance to a known phytoalexin. The ABC transporters BcatrB in *Botrytis cinerea* and GpABC1 in *Gibberella pulicaris* provide tolerance to the host plant's phytoalexins, resveratrol and rishitin, respectively [48],[51]. A *BcatrB* mutant is reduced in pathogenicity on grape leaves [48], while a *GpABC1* mutant is essentially nonpathogenic on potato [51]. Few examples of fungal MFS transporters involved in tolerance to phytoalexins exist, although a MFS transporter from *N. haematococca* is able to confer an increase in pathogenicity on pea when placed in a nonpathogenic pea isolate [52]. This MFS transporter–encoding gene, termed *PEP5* for pea pathogenicity, is induced upon exposure to the pea phytoalexin pisatin [53]; however, the mechanism by which PEP5 contributes to virulence remains unknown.

Fungicide resistance is also due to ABC and MFS transporters. Several transporters in the agriculturally relevant fungi *B. cinerea* and *Mycospharella graminicola*, as well as the model filamentous fungus *A. nidulans*, have been reviewed recently [54]. The ABC transporter BcatrB of *B. cinerea* is also able to provide resistance to the fungicides fenpiclonil and fludioxonil in addition to resveratrol, as referenced above [48],[50].

## Transporters Involved in Mammalian Virulence

The most common human pathogenic fungus, *C. albicans*, has a total of 21 ABC transporters and 85 MFS transporters encoded within its genome (Table 2). A single ABC transporter that is required for full virulence has been identified in *C. albicans*. This transporter, MLT1, is a vacuolar transporter and a member of the ABCC/MRP family of proteins. Mutants of this transporter are severely defective in invasion of the liver and pancreas in a mouse peritonitis virulence assay and cause less hepatic tissue damage [55]. The exact substrate(s) of this efflux pump are not currently known; however, MLT1 is similar to the bile pigment transporter BPT1 in *S. cerevisiae*, which is involved in sequestering unconjugated bilirubin and glutathione conjugates in the vacuole [56],[57], suggesting that MLT1 is involved in resistance to similar compounds.

## ABC Transporters in Clinical Multi-Drug Resistance

Although not directly involved in virulence, the ability to provide resistance to antifungal compounds does provide a “colonization” advantage to the fungus and, therefore, merits further discussion.

A number of transporters that confer resistance to members of the antifungal class of triazoles have been described. These include the ABC transporters CDR1 and CDR2 in *C. albicans*; AFR1 in *C. neoformans*; ABC1 in *C. krusei*; and CgCDR1, PDH1 (also referred to as CgCDR2), and SNQ2 in *C. glabrata* [58]–[63]. These transporters are usually able to provide cross-resistance to the antifungal class of triazoles (fluconazole, itraconazole, ketoconazole, voriconazole); however, studies suggest they are not responsible for echinocandin resistance or resistance to the antifungal protein histatin 5 [64],[65]. These transporters are closely related and all belong to the PDR family, which includes the well-characterized PDR5 transporter in *S. cerevisiae*. The PDR family is one of the largest families of ABC transporters in fungi and is overrepresented when their numbers are compared to other organisms, i.e., there are five and nine ABCG/PDR transporters in humans and *Caenorhabditis elegans*, respectively [66],[67]. Although initially identified for conferring an MDR phenotype, studies of CDR1 and CDR2, and most likely the orthologous genes in other pathogenic fungi, have demonstrated that their physiological function is the transport of phospholipids and steroids across the membrane [68]–[70].

Of the transporters involved in fluconazole resistance in medically important fungi, CDR1 and CDR2 are the most studied. Despite their high degree of amino acid similarity, there are functional differences between the two. Mutants of *CDR2* retain a wild-type level of resistance to fluconazole; however, in a double mutant (*ΔCDR1*/*ΔCDR2*) the strain is more susceptible to fluconazole than either single mutant [71]. Other differences in function have been identified, suggesting that the two ABC transporters have separate, but overlapping, roles in the fungus [72]. In addition, there is allelic variation of the genes. Comparison of the CDR2 alleles uncovered two point mutations in equal frequency of an allele in transmembrane helix 12 that were involved in substrate binding and function [73]. Most strains were heterozygous for these *CDR2* genes, and further phylogenetic analysis suggested that as many as 33 codon changes between the two alleles may be selectively advantageous [73]. Differences in transmembrane domains are significant, as evidence builds that they are responsible for substrate specificity. Most ABC transporters have been demonstrated to have multiple substrates; thus, it is postulated that substrate binding does not occur in a specific active site, but rather in an active pocket able to accommodate a variety of structurally different compounds [74],[75]. Several amino acids are conserved among these transmembrane helices of fluconazole PDR transporters, where the most conserved helices are 6 and 12.

## MFS Transporters in Clinical Multi-Drug Resistance

In addition to ABC transporters, a number of MFS transporters also are responsible for increased resistance to fluconazole. The two best characterized are MDR1 (formally BEN^r^) and FLU1 in *C. albicans* [76]–[79], and homologs of *MDR1* have been implicated in fluconazole resistance in *C. dubliniensis* [80],[81] and *C. tropicalis* [82]. Unlike the ABC transporters, the resistance conferred by MFS transporters is more specific for fluconazole than other triazoles, although other substrates have been identified [76],[80]. Of particular importance for MDR1 function is transmembrane domain 5, which harbors a conserved motif important for drug:H^+^ translocation (Figure 1) [78]. Other MFS transporters (TMP1 and TMP2) confer increased resistance to a number of unrelated antifungal drugs in *C. albicans* [83]. It should be noted that other transporters may be involved in resistance to antifungals. For example, the Sec14p family member PDR16 transporter in *C. albicans* is upregulated in fluconazole-resistant clinical isolates, and confers a 2-fold increased resistance to the azole antifungal [84].

## Expression of Efflux Pumps in the Multi-Drug Resistance Phenomenon

Pathogenic isolates are able to develop resistance to prescribed antifungal treatment rapidly [85],[86]. While other factors contribute to this increase in resistance, one reason is the increase in transcription of drug transporters [85], [87]–[91]. A shared 22–base pair sequence in the promoters of *CaCDR1* and *CaCDR2*, termed the drug responsive element, serves as the binding site of the Zn(2)-Cys(6) finger transcription factor TAC1 [92],[93]. Although azole-resistant isolates have been demonstrated to sometimes carry an extra copy of the chromosome that has *CDR1* and *CDR2*, the transcript levels did not increase [94], confirming other studies that suggest the increase in transcription is due to either the promoter or *trans*-acting factors of the *CDR* genes [95]. Indeed, a second chromosomal rearrangement was identified where resistant isolates frequently harbor duplications of the chromosomal region in which TAC1 resides [96]. Recently, the analogous transcription factors of TAC1 in *C. glabrata* (CgPDR1 and CgPDR3) have been demonstrated to bind the substrate, promoting the expression of the efflux pumps under its control [97]. Another zinc finger transcription factor (MRR1) is responsible for the overexpression of the MFS transporter encoding gene *MDR1* [98]. Two point mutations in MRR1 of azole-resistant isolates were identified as rendering the transcription factor constitutively active [98],[99]. Unlike MDR1, expression of *FLU1* in clinically resistant isolates is not increased, and therefore FLU1 is not believed to contribute significantly to resistance to fluconazole [79].

A major difference in the mutations of the major transcription factors that confer fluconazole resistance is the heritability of the resistance phenotype. The *CDR1*/*CDR2* overexpressing *C. albicans* isolates require the *TAC1* fluconazole-resistant transcription factor to be homozygous, as heterogygous isolates retain wild-type levels of fluconazole susceptibility [100]; however, the nature of the point mutations of MRR1, which render the transcription factor constitutively active, functions in a semi-dominant manner, as a single copy of the fluconazole-resistant mutant *MRR1* increases fluconazole resistance [98]. Other transcription factors involved in *CDR1*, *CDR2*, and *MRR1* overexpression have been identified, but their role in clinical resistance is less understood. These transcription factors include CaNdt80 in *CDR1* overexpression [101], and Cup1 and Mcm1 in *MDR1* overexpression [102],[103]. Recent studies also describe an increase in mRNA stability of *CDR1* transcripts in fluconazole-resistant *C. albicans* isolates that results in an increase in the number of CDR1 transporters [104].

## Conclusions

The role of efflux pumps, in particular ABC and MFS transporters, in fungal virulence cannot be understated. These transporters contribute to pathogenesis by 1) transporting HSTs and mycotoxins outside of the cell, 2) removing host-derived compounds that would otherwise inhibit the fungus, and 3) providing resistance to clinical antifungals. This concept is further supported by the vast amount of substrate redundancy already evidenced with the few transporters that have been extensively studied. Considering *S. cerevisiae* and *C. albicans* have relatively few ABC and MFS transporters compared to filamentous fungi, and the scope of research already conducted on these important yeast superfamilies, it appears there is much more to be discovered in other fungi.

The redundant nature of the transporters creates difficulty in characterization of the efflux mechanisms, as single deletion mutants sometimes retain a wild-type phenotype, and it is only through multiple mutations of transporters or expression in heterologous systems that the function of the transporters becomes apparent. Although a daunting endeavor, understanding all of the contributing efflux factors of a given antimicrobial will provide a means to circumvent efflux transporters.
